# Organizational strategies for managing COVID-19 survivors who return for care

**DOI:** 10.1017/ice.2020.456

**Published:** 2020-09-09

**Authors:** Sarah D. Haessler, Hilary M. Babcock, Mary K. Hayden, Trevor Van Schooneveld, David J. Weber, Anurag Malani, Rekha Murthy, Judith A. Guzman-Cottrill, Sharon B. Wright, Clare Rock, Corey A. Forde, Latania K. Logan, David K. Henderson

**Affiliations:** 1 UMass Medical School-Baystate, Springfield, Massachusetts; 2 Washington University School of Medicine, St. Louis, Missouri; 3 Rush University Medical Center, Chicago, Illinois; 4 University of Nebraska Medical Center, Omaha, Nebraska; 5 University of North Carolina at Chapel Hill, Chapel Hill, North Carolina; 6 St. Joseph Mercy Health System, Ann Arbor, Michigan; 7 Cedars-Sinai, Los Angeles, California; 8 Oregon Health and Science University, Portland, Oregon; 9 Beth Israel Deaconess Medical Center, Boston, Massachusetts; 10 Johns Hopkins University School of Medicine, Baltimore, Maryland; 11 Queen Elizabeth Hospital, Christ Church, Barbados; 12 National Institutes for Health, Bethesda, Maryland

As the coronavirus disease 2019 (COVID-19) pandemic has evolved, survivors are now returning for care at hospitals and ambulatory sites, creating confusion and uncertainty for the frontline staff regarding proper management of these patients. Although the US Centers for Disease Control and Prevention (CDC) provides clear guidance on both symptom-based and test-based strategies for discontinuation of transmission-based precautions,^1^ many healthcare workers lack confidence that the symptom-based strategy of 10 days of isolation is long enough to assure that the patient is not infectious. Thus, some staff are uncomfortable managing a patient using standard precautions, especially if the patient has undergone repeat testing and remains positive for severe acute respiratory coronavirus virus 2 (SARS-CoV-2) as indicated by real-time polymerase chain reaction (RT-PCR) testing. For hospitals that routinely test all admissions, should patients who have recovered from COVID-19 be included? And how should these patients be managed when it comes to preprocedure testing?

In the absence of definitive guidance or good-quality published evidence, healthcare epidemiologists must use the best available evidence to make practical and rational decisions. Making these decisions often requires acknowledgement and appropriate balance of 3 important goals: (1) keeping patients and employees safe from harm, which includes avoiding unnecessary delays in care for COVID survivors; (2) ensuring that resources, such as personal protective equipment (PPE) and testing reagents and materials, remain available for the highest-risk situations; and (3) ensuring that staff feel comfortable coming to work and caring for patients. To accomplish these goals, explicitly communicating what is known and not known about the epidemiology, biology, transmission, and mitigation strategies for COVID-19 often becomes necessary while acknowledging that new information will continue to emerge and that guidance may change as a result.

The goal of initial SARS-CoV-2 RT-PCR testing is to identify patients who have active COVID-19 for clinical decision making and to identify patients who require transmission-based precautions to prevent the spread of infection. Once a patient has a positive RT-PCR result, 2 options exist for discontinuing transmission-based precautions: (1) test-based strategies, which generally require 2 negative tests at least 24 hours apart to end isolation and (2) symptom-based strategies, which discontinue isolation typically 10 days after symptom onset and resolution of fever for at least 24 hours without antipyretics and improvement in respiratory symptoms. We now have accumulated evidence that SARS-CoV-2 remains detectable by RT-PCR for an average of 3 weeks^2^; however, we also have evidence demonstrating that, for uncomplicated, recovering COVID-19 patients, replication-competent virus cannot be cultured >9–11 days after onset of illness.^2–4^ The CDC’s unpublished data indicate that the statistically estimated likelihood of recovering replication-competent virus approaches zero by 10 days, which is the basis for their symptom-based strategy for discontinuing isolation precautions.^1,5^ In May 2020, the Korean CDC published evidence that patients with COVID-19 who had late positive (ie, “re-positive”) RT-PCR testing after interim negative tests did not transmit the infection to close household contacts.^6^ Of 285 monitored cases with 790 contacts, no case was found that was newly infected solely from contact with a “re-positive” case. They obtained respiratory samples from 108 of these patients and were unable to culture viable virus from any of them. For 23 patients, blood samples were also obtained, and all were positive for neutralizing antibodies. These data support the US CDC’s symptom-based strategy by demonstrating that close contacts of patients with prolonged detection of SARS-CoV-2 by PCR did not become infected and, thus, that these patients were not contagious despite having repeated positive RT-PCR results.

Accumulating evidence suggests that symptom-based strategies are sound for uncomplicated cases of COIVD-19, but data evaluating immunocompromised patients, such as solid organ transplant patients and oncology patients, and those with severe illness are scarce. A recent publication from The Netherlands,^7^ which included primarily ICU patients found infectious viral shedding in only 23 of 129 patients (18%), with a median duration of shedding of 8 days after symptom onset. Interestingly, viable virus was detected in only a small number of patients, with the longest detected at 20 days. The likelihood of detected viable virus was <5% after day 15.

In light of this evidence, we suggest that most previously positive patients who return for care and who have met the CDC’s symptom-based strategy for discontinuation of transmission-based precautions need not be retested prior to readmission or prior to a procedure. Furthermore, they can be safely managed using standard precautions. For the subgroup of complicated patients who may have the potential to shed live virus for longer, such as those who required intensive care or are severely immunocompromised, a more nuanced approach may be needed. In these cases, the CDC guidance suggests that patients should be isolated for up to 20 days. The above data are reassuring that even in severely ill patients the more conservative time period ensures that no infectious virus is present and even those who persistently test positive could be removed from isolation. Test-based strategies may be considered to help end isolation earlier than 20 days. However, from a practical perspective, it is challenging to simultaneously implement test-based and time-based strategies effectively. One strategy is to discourage repeat testing and base decisions solely on time-based criteria for most patients.

Ensuring that frontline staff understand the rationale for institutional recommendations, feel comfortable with the evidence, are willing to follow the guidance, and understand how to manage these patients requires an understanding of the barriers that often hamper the acceptance and roll-out of policies. Frequently modified written policies based on the most up-to-date evidence and that are developed with the input of multiple stakeholders will promote ownership in key areas of the organization. Education of nursing and medical staff through departmental leadership and staff meetings will be required to gain buy-in. Concerns should be addressed regarding areas that may be considered “higher risk,” such as procedural areas, oncology wards, and treatment centers. Especially in situations that are novel and fraught with uncertainty or fear, such as the COVID-19 pandemic, enlisting the trust of hospital staff in hospital leadership is key to success. When resistance is met, a system for the real-time escalation of individual cases to well-informed medical leadership (eg, chief medical officer, chief quality officer, or healthcare epidemiologist) should be in place (1) to allow hospital leadership to listen carefully to staff concerns, (2) to provide simultaneous education of staff and resolution of the challenging situation, (3) to provide appropriate management and placement of the patient, and (4) to provide clarity to team members, families, and the patient, thereby engendering further trust.
